# Supplementary material for: Social anxiety influences the stress-buffering potential of social presence: Evidence from cardiovascular and affective reactivity under stress
Source: PLoS One. 2025 Jun 3;20(6):e0325303. doi: 10.1371/journal.pone.0325303 (PMC12132970; doi:10.1371/journal.pone.0325303)
Supplement: Attachment — Submitted filename: R1_Response_Letter.docx [file pone.0325303.s002.docx]

We would like to thank both reviewers for their positive evaluation of the manuscript and for their comments. In this letter we address each reviewer’s comment, and point to the changes we made in the revised manuscript according to their suggestions.

## **Reviewer #1**

*1. There were spots throughout the introduction where a citation was needed and there isn’t one. For example, the definition of social anxiety. Please add more citations throughout the introduction to back up claims.*

We have added an operational definition of social anxiety in the Introduction (page 5 of the revised manuscript, highlighted in yellow) and included new citations where appropriate (citations #19 and #20 in the revised manuscript). The new paragraph now reads: “*Social anxiety can be defined as the tendency of individuals to be wary of others and to experience discomfort in social interactions. Low levels of social anxiety are normally experienced in social interactions, especially when new and/or unexpected. In a minority of the population affected by a social anxiety disorder, this experience of negative emotions is far more intense and accompanied by strong physiological arousal and leads to a significant impact on their daily functioning and well-being.*”

*2. Was there a directional hypothesis about the relation between social anxiety and stress reactivity in each group. Different possibilities were discussed but there did not appear to be a specific hypothesis.*

No, we did not have a strong hypothesis regarding the direction of the relationship between social anxiety and stress reactivity. We discussed in the Introduction both alternative possibilities, to disclose the exploratory nature of this specific part of our study (Page 7, Introduction section).

*3. Were those in the stranger condition in romantic relationships? I understand that they were paired with strangers but did they have relationships outside of this study? If not, there might be systematic differences between the groups. There might be differences in how people perceive support from others based on their own relationship experiences so this might be listed as a limitation if they were not in relationships.*

Regarding the reviewer’s observation about relationship status within our sample, we acknowledge that only 50% of the participants in the Stranger group (N = 11) were involved in a romantic relationship during the study period, while the remaining participants were not. Following the reviewer’s valuable suggestion, we have explicitly incorporated this limitation in the conclusions section of our manuscript, which now reads: “*A fourth limitation is represented by the fact that only half of the participants included in the Stranger condition were in a romantic relationship at the time of the research. At present, we can not rule out that being in a romantic relationship might have an impact even when facing a stressor together with a stranger providing support. In future studies, this feature should be controlled in order to evaluate possible differences in stress reactivity that could depend entirely on being or not in a romantic relationship, independent from having support when facing a stressor.* ” (Page 20 of the revised manuscript, highlighted in yellow)

*4. Was there rationale for always having the target participant be female? This was not set up in the introduction and came as a surprise*

We included only female participants to minimize the confounding effect of the known gender differences in emotional reactivity. We report this information in the conclusions highlighting that this poses a constraint on the generalizability of our findings: “*This choice was made to minimize the potential confounding effects of the gender differences that exist in affective and psychophysiological reactivity, and in line with previous research showing that stress buffering on autonomic reactivity is larger in women. Nonetheless, this warns against a generalization of these effects also to the male population.*” (Page 19 of the revised manuscript, highlighted in yellow)

*5. What was the reliability of the STAI and SIAS scales in the current sample?*

We computed the Chronbach’s alpha for both measures and found excellent reliability. For SIAS the Chronbach’s alpha was 0.91, while for STAI it was 0.94. We reported this information in the revised manuscript (pages 12 and 14 of the revised manuscript, highlighted in yellow)

*6. Did the two groups differ on any demographic variables? Baseline statistics should also be reported, perhaps in a table.*

The groups did not differ in terms of age. This information is now reported in the manuscript (page 8 of the revised manuscript, highlighted in yellow).

*7. Were there any differences in stress reactivity or anxiety based on the gender of the supporter?*

We tested this possibility by refitting the model including also the gender of the supporter as a predictor and compared the original model with this second model with a likelihood-ratio test (LRT). The significance of the LRT would indicate that the additional variable (gender of the supporter) would increase the explained variance in the data.
Results indicate that the gender of the supporter does not contribute to explaining neither stress reactivity nor anxiety. The reviewer can find the results of these analyses below:

LRT result for anxiety

Models:

mod_state_anxiety: stai_s ~ time * group + (1 | dyad)

mod_state_anxiety_2: stai_s ~ time * group * gender_alt + (1 | dyad)

npar	AIC	BIC logLik deviance Chisq Df Pr(>Chisq)

mod_state_anxiety	10 1179.4 1210.2 -579.70	1159.4

mod_state_anxiety_2	18 1187.0 1242.4 -575.53	1151.0 8.352 8	0.3999

LRT result for stress

mod_stress: stress ~ time * group + (1 | dyad)

mod_stress_2: stress ~ time * group * gender_alt + (1 | dyad)

npar	AIC	BIC logLik deviance Chisq Df Pr(>Chisq)

mod_stress	10 681.97 712.66 -330.98	661.97

mod_stress_2	18 694.20 749.44 -329.10	658.20 3.768 8	0.8774

*8. I am struck by the difference in the physiological and psychological results. I agree with the authors’ proposal that the emotional concern might outweigh the safety signal of their partner or that performing in front of a partner is an added evaluative pressure. I have a potential third option as well. The TSST is a fairly negative experience and I’m wondering if it is possible that the target participant feels safer to express negative feelings in the presence of their significant other.*

The third option offered by the reviewer is very interesting. We included it in the conclusion of our revised manuscript as a potential alternative interpretation and suggested future experimental design that could help clarify this issue. The new paragraph now reads: “*Future investigation should focus on designing more complex manipulation of social support. Indeed, a potential alternative interpretation for finding that participants performing in front of their partner report higher anxiety than those performing in front of a stranger, is that the former could have felt more comfortable in disclosing their levels of negative emotions due to their partner's presence. Explicitly manipulating the behavior of the partner could help clarify when its presence can act as a source of emotional buffer or as an additional source of psychological distress.*” (page 20 of the revised manuscript, highlighted in yellow).

## **Reviewer #2**

*1. The Manuscript needs careful English proofreading because there are some shortcomings. For instance, the article “the” is sometimes missing in front of nouns, the message in some paragraphs is not clear enough. It looks like the first part was written by one author with a greater command of the English language, and the rest of the paper was written by someone else. The numerous grammar errors made this a difficult paper to read. It was strange to see the authors refer to tables that were not submitted. I was unable to find any supplementary material to the submission, so I think this was truly omitted by the authors. Please read the manuscript carefully.*

*3. The research is well based on science and the results are in agreement with the theoretical part. From my point of view, the paper is original and the topic addressed brings added value to the specialized literature regarding the anxiety. The paper is well written and easy to read.*

We sincerely appreciate the reviewer thoughtful feedback, particularly the kind words regarding the paper's clarity and readability in comment #3. The positive remarks are truly encouraging.

With regards to the suggested proofreading, we asked our university proofreading services and revised the manuscript accordingly.

We would also like to address a potential misunderstanding concerning supplementary materials. We apologize if this caused any confusion, but we did not include any supplementary materials in our original submission. This explains why you were unable to locate them during your review.

Thank you once again for your valuable input and the time you've dedicated to reviewing our work.

*2. It would be very useful to add in the "Introduction" section the purpose, objectives and hypothesis of the research. I consider that a weak point of the paper is that the authors did not show the novelty of the paper compared to other works. That is why, I consider that the introduction should specify the novelty of the paper compared to other papers published in this area.*

*5. The Literature Review and the conclusions sections cannot be misssing from the paper*

In the revised version of the manuscript, we have now better distinguished the hypotheses and the goals of the manuscript, adding a new subsection in the Introduction section titled “Research objectives” (page 6 in the revised manuscript, highlighted in yellow). Following comment #5, we have now separated the discussion from the conclusions sections of our manuscript to improve clarity according to the reviewer’s suggestion.

*6. In my opinion, the results from the Statistical Analysis section, which presents three models with dependent variables and random effects, should be presented in a table. Here, the authors should apply the Hausman and VIF tests (see:* [*https://doi.org/10.1371/journal.pone.0283277*](https://doi.org/10.1371/journal.pone.0283277)*).*

*7. Authors must refer to the software used for econometric analyses: STATA, EViews, SPSS etc.*

*8. The authors must also show the values of the adjusted R-square, which is more relevant in the methods used in this paper.*

We reported in our revised manuscript that all the statistical analyses were performed using R 4.1.2 (page 11 in the revised manuscript, highlighted in yellow).

Following the reviewer’s suggestion, we reported the results for each model in tables (Table 1, Table 2 and Table 3) and reported the additional information requested by the reviewer (R^2^, VIF). With regards to VIF for all three regression models, VIF was 1, suggesting no collinearity.

*4. The research is well based on science and the results are in agreement with the theoretical part. The model applied to the analyzed data is correctly used in the analysis undertaken, it is a strength point of this paper.*

*9. The authors applyed the regression method, then they must apply the following tests: the authors must to apply an econometric method like regression or panel with fixed effect estimation or the random effect estimation (see for instance, Baltagi (2008), Hsiao (2014) and Andre B et al. (2015)). Besides, the corresponding tests to determine which is the best method of estimation is needed (see the Hausman test, the Breusch and Pagan (1980)´s Lagrange multiplier, the F test for fixed effects to test whether all unobservable individual effects are zero); The authors talk about the relationship between these variables, however they do ot support the empirical evidence providing panel cointegration tests that are crucial (see for instance Kao (1999) panel data cointegration test, the Pedroni (1999, 2004) panel data cointegration test or the Westerlund (2005) panel data cointegration test, among others).*

*10. In the same time the authors must present the results of Dumitrescu-Hurlin test.*

*11. In the same time, the authors should test the validity of the model with the help of residual analysis using the Jarque-Berra test.*

We appreciate the Reviewer's positive feedback on our statistical approach in comment #4, where they found our analyses appropriate for the study’s goal and highlighted them as a strength of the paper.
Regarding the suggested methods in comments 9-11 (Hausman test, panel cointegration test, Lagrange multiplier test, Dumitrescu-Hurlin test, and Jarque-Berra test), we acknowledge that these econometric methods have an established value in financial and economic research contexts.

However, these methods are not commonly employed in experimental psychology and psychophysiological research. We have opted to maintain our current statistical framework to ensure alignment with methodological standards in our field, thereby preserving readability and potential impact for our target audience. We believe this approach best serves the paper's objectives while adhering to rigorous methodological standards.
